# Synergistic prevention and reparative effects of sesquiterpene farnesol in a rabbit model of surgical resection-induced osteoarthritis

**DOI:** 10.1063/5.0129530

**Published:** 2023-01-13

**Authors:** Chun Yu Chen, Shyh Ming Kuo, Guan Xuan Wu, Shan Wei Yang

**Affiliations:** 1 Department of Electrical Engineering, I-Shou University, Kaohsiung City, Taiwan; 2 Department of Orthopedics, Kaohsiung Veterans General Hospital, Kaohsiung Veterans General Hospital, Kaohsiung City 81346, Taiwan; 3 Department of Biomedical Engineering, I-Shou University, Kaohsiung City, Taiwan

## Abstract

Articular cartilage may regenerate poorly after injury or during aging. *In vitro*, farnesol can modulate extracellular matrix synthesis and restore chondrocyte phenotypes by increasing type II collagen (COL II) and glycosaminoglycan (GAG) production. Here, we evaluated farnesol's preventive and reparative effects against osteoarthritis (OA) *in vivo*. We induced OA in rabbits through resection of the lateral collateral ligament and meniscus. After 2 weeks, the affected limb was treated with 0.5 ml of 0.4 mM farnesol, hyaluronan (HA) nanoparticle-encapsulated 0.8 mM farnesol (Farn/HA), or HA nanoparticles intra-articularly. After 2 and 6 treatment weeks, synovial inflammatory cytokine levels were analyzed. We also removed the entire joint cartilage from lateral femoral condyles for histological investigation. The half-maximum inhibitory concentration of farnesol was 0.5 mM. Farn/HA had relatively low cytotoxicity showing cells remained viable after being treated with 1 mM a concentration Farn/HA. Untreated lateral condyle exhibited extensive wear. By contrast, 0.4 mM farnesol or 0.8 mM Farn/HA led to a relatively transparent and bright appearance. After 2 and 6 treatment weeks, farnesol, particularly 0.8 mM Farn/HA, reduced matrix metalloproteinase 1 and 13 levels considerably. Therefore, 0.8 mM Farn/HA, which enabled slow drug release, demonstrated the highest anti-inflammatory and OA preventive effects. After 6 treatment weeks, farnesol also promoted COL II and GAG synthesis and, thus, aided healing.

## INTRODUCTION

Articular cartilage has a low chondrocyte density (1%–5% of the total volume)^1^ and avascular nature,^2^ and it lacks lymphatics;^3^ therefore, it has poor regeneration capabilities. After injury and during aging, articular cartilage may be unable to repair itself. After damage due to injury or overuse, weight-bearing biomechanisms of articular cartilage become altered, leading to progressive worsening of the condition and inducing osteoarthritis (OA).^4^ The pathophysiology of articular cartilage deterioration involves increase in its water content and alterations and eventual decreases in proteoglycan levels in the extracellular matrix (ECM).^5^

After the alteration of the cartilage texture, a joint begins degenerating; the underlying mechanism may involve inflammatory changes in the synovium followed by the development of synovial hyperplasia or hypervascularity, remodeling of the subchondral bone, the formation of lytic lesions with sclerotic edges, and the proliferation of osteophytes, gradually affecting the appearance of the joint with varus or valgus deformation.^6^ Surgical intervention is required when symptoms can no longer be controlled using medication and rehabilitation therapy at the advanced stage. In younger patients with degeneration in only a single compartment, a proximal tibial osteotomy can be performed for contrary correction, thus preventing painful loading.^7^ In older patients with single-compartment degeneration, unicompartmental arthroplasty may be performed.^8^ However, total knee arthroplasty is required for severe degeneration involving two or more compartments.^9^ Tissue engineering for cartilage regeneration and repair and to avoid surgery has been a research focus.

Chondrocytes remodel the ECM by synthesizing, maintaining, and degrading in response to signals from cytokines, inflammatory mediators, and matrix fragments.^10^ In a healthy condition, articular chondrocytes are typically quiescent and highly differentiated; they are phenotypically stable and normally synthesize and maintain resilient ECM constituents principally composed of type II collagen (COL II) and aggrecan. Phenotypically unstable chondrocytes undergo dedifferentiation, resulting in the production of a substantially different protein, which integrates into the ECM, leading to inferior mechanical properties.^11^ Numerous changes occur in dedifferentiated chondrocytes, including a considerable distortion in cell morphology, decreased expression of chondrogenic transcription factor SOX9, and suppressed production of the cartilage-specific matrix proteins, such as COL II and aggrecan. Cell metabolism alteration considerably increases the synthesis of COL I and type X collagen (COL X). In chondrocytes, phenotypic instability or morphologic and volume changes, which occur before considerable cartilage degeneration, might be a main feature of OA development.^11,12^

Autologous chondrocyte implantation (ACI) involves the implantation of autologous chondrocytes cultured *in vitro* over the cartilage defect. In 1994, Brittberg *et al.* described this technique as one of the first tissue engineering techniques for articular cartilage regeneration.^13^ Currently, ACI is performed in two stages. The first step is to take a cartilage biopsy (weight 200–300 mg) from an area of the affected joint that is not in contact with weight-bearing activity. In the laboratory, enzymatic digestion is used to isolate chondrocytes from cartilage tissue. Then, monolayer cultures are used to expand them. Despite promising results,^14^ ACI has limitations due to the limited availability of cells and the possibility of dedifferentiation during the expansion of chondrocytes *in vitro*. Moreover, implanting and adhering to definite lesions is the most challenging aspect of this process. It is anticipated that an alternative treatment that does not require surgical placement and fixation of the defect will be the way of the future. Instead, the cells' own healing ability will be stimulated to achieve therapeutic results by intra-articular injection of agents. Nanoparticles (NPs), defined in size range of 1 ∼ 100 nm, have high loading capacity and specific targeting to cells due to their size effect and intracellular uptake. Nanoparticles used for drug delivery are sub micrometer colloidal particles prepared from biocompatible and biodegradable materials. HA, a natural glycosaminoglycan, is biodegradable, biocompatible, and hydrophilic. HA is also a component of the extracellular matrix in cartilage tissue. Recently, HA is used in various drug delivery methods/materials to encapsulate active drugs/compounds or genes. We have successfully fabricated nano-sized HA particles with drugs in an aqueous-phase environment with anti-tumor activities using a high-voltage electrostatic field system under experimentally controllable parameters.^15^ In this study, the farnesol was aggregated with HA nanoparticles to prepare Farn/HA nanoparticles using this system, and the prevention and reparative effects of pure farnesol and Farn/HA nanoparticles on surgical-induced OA were investigated.

Farnesol is an organic 15-carbon sesquiterpene compound produced by *Candida albicans*; it exhibits antioxidant,^16^ anti-inflammatory,^17^ antimicrobial,^18^ and tumor-related apoptosis-inducing properties.^17^ Ku *et al.* demonstrated that farnesol downregulates essential inflammatory cytokines, such as interleukin (IL) 1β, IL-6, and tumor necrosis factor alpha (TNF-α), *in vivo*.^19^ Farnesol can modulate connective tissue and ECM synthesis, which is required for wound healing^20^ and rotator cuff repair.^21^ Cartilage reconstruction is challenging because cartilage has poor intrinsic defect-repairing abilities. Studies have identified that in chondrocytes, phenotypic instability occurs with changes in the cell volume and morphology before considerable cartilage degeneration and loss, which might be essential for indicating the early stages of cartilage loss.^12^ Wu *et al.* investigated the effect of farnesol on IL-1β-induced dedifferentiated chondrocytes. The authors suggested that farnesol restores the phenotype of the chondrocytes and salvages their ECM COL II and glycosaminoglycan (GAG) production capacity *in vitro.*^22^ Therefore, this study investigated whether intraarticularly administered farnesol could prevent further cartilage degeneration in a rabbit model with anticipated cartilage degeneration, and further investigated if they have a therapeutic effect.

## RESULTS

**FIG. 1.**
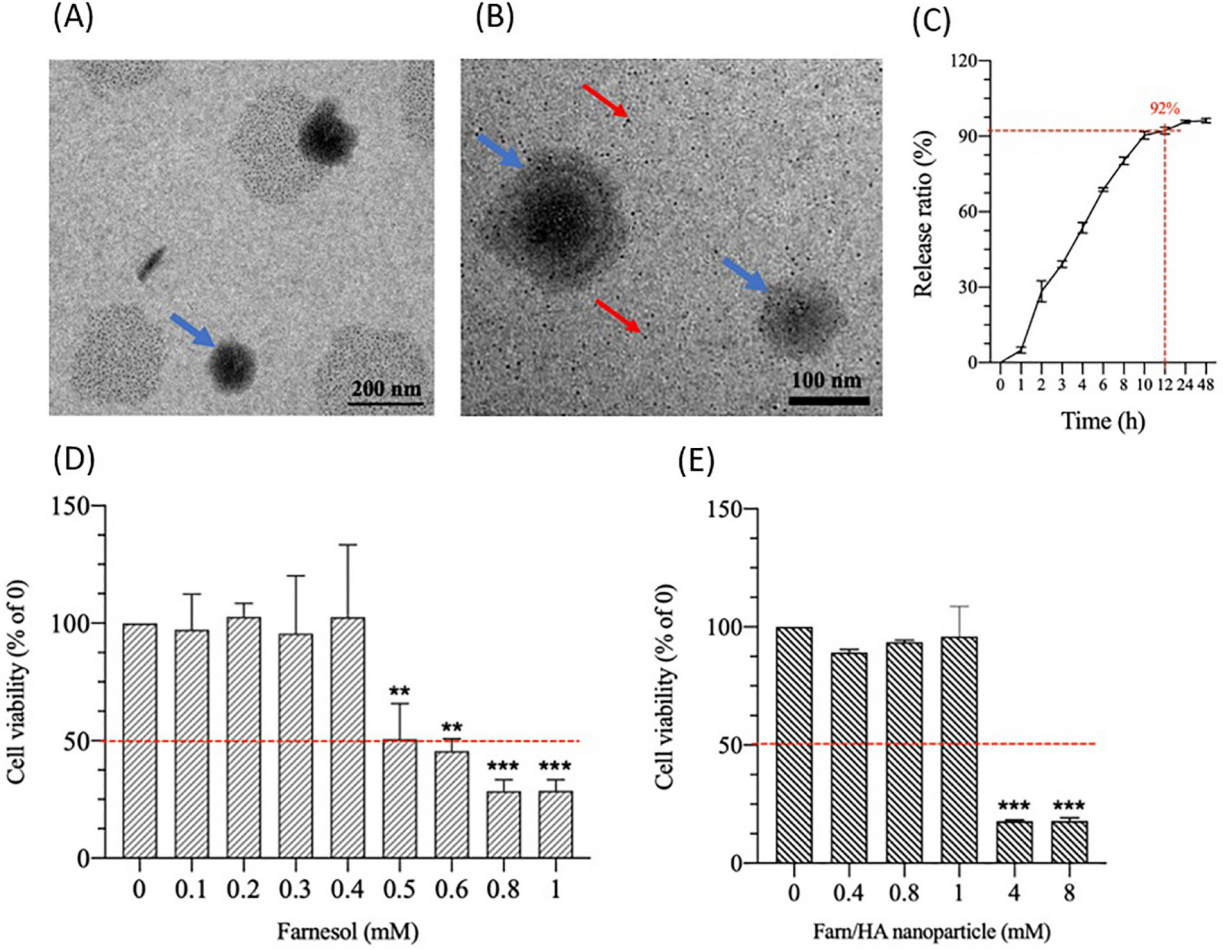
Biological characteristics of HA and Farn/HA nanoparticles: (a) TEM images of HA nanoparticles and (b) TEM images of Farn/HA nanoparticles. HA nanoparticles were labeled with red arrow and Farn/HA nanoparticles with the blue arrow. (c) Release profile of farnesol from Farn/HA. Approximately 92% of the farnesol was released after 12 h of incubation. Cell viability after exposure to (d) farnesol alone and (e) Farn/HA on the basis of the MTT assay. As compared with 0 mM farnesol or 0 mM Farn/HA nanoparticles, ** p < 0.01 and *** p < 0.001 (n = 3).

Transmission electron microscopic (TEM) images revealed that the HA nanoparticles and Farn/HA were successfully fabricated under high electrostatic field strength at certain settings [Figs. 1(a) and 1(b)]. The HA nanoparticles, produced first in the preparation system, had a relatively homogeneous diameter of approximately 5 nm. As the treatment progressed, Farn/HA gradually formed because of hydrophobic and hydrophilic interactions between the HA nanoparticles and farnesol molecules in the electrostatic field environment, yielding HA nanoparticle-farnesol aggregates (i.e., Farn/HA).^22^ At the treatment temperature (20–25 °C), which enabled the mobility of HA nanoparticles and farnesol molecules, the structure of the formed Farn/HA was nonuniform; initially (i.e., in the short-term), it was relatively loose, and as the treatment progressed, it became denser. The experiments on and mechanisms underlying the preparation of Farn/HA are not covered in this study.

The Farn/HA had a mean diameter of approximately 110.5 ± 27 nm. The EE of farnesol in Farn/HA was approximately 91%, and approximately 92% of the farnesol was released after incubation for 12 h in a phosphate-buffered solution [Fig. 1(c)]. The *in vitro* effects of farnesol and Farn/HA on chondrocyte viability were examined using the MTT assay [Fig. 1(d)]. We detected nonsignificant reductions in chondrocyte viability after 24 h of incubation with <0.4 mM farnesol. The half-maximum inhibitory concentration (IC_50_) of farnesol in chondrocytes was approximately 0.5 mM. Chondrocytes demonstrated a considerable reduction in viability only after exposure to Farn/HA at high concentrations [4 and 8 mM; Fig. 1(e)]. We previously demonstrated that encapsulation of a drug in HA nanoparticles can both reduce its IC_50_ and enable its slow release, thus enhancing its therapeutic effects.^19^

The half-maximum inhibitory concentration (IC50) of farnesol on the primary-cultured chondrocytes was 0.5 mM in this study; thus, we chose 0.4 mM farnesol, in order not to exert more toxicity and impair the function of primary-cultured chondrocytes. The farnesol concentration increased to 0.8 mM in the Farn/Ha nanoparticles (2-fold increase in 0.4 mM farnesol) for the slow-release behavior of farnesol from Farn/HA nanoparticles to assure enough amount of farnesol; importantly, this 0.8 Farn/HA nanoparticles concentration did not significantly exert toxicity to the chondrocytes [Figs. 1(d) and 1(e)]. According to the result, we chose these two concentrations to survey the repair and prevention effects of farnesol on the OA in the animal study.

### Gross morphology

**FIG. 2.**
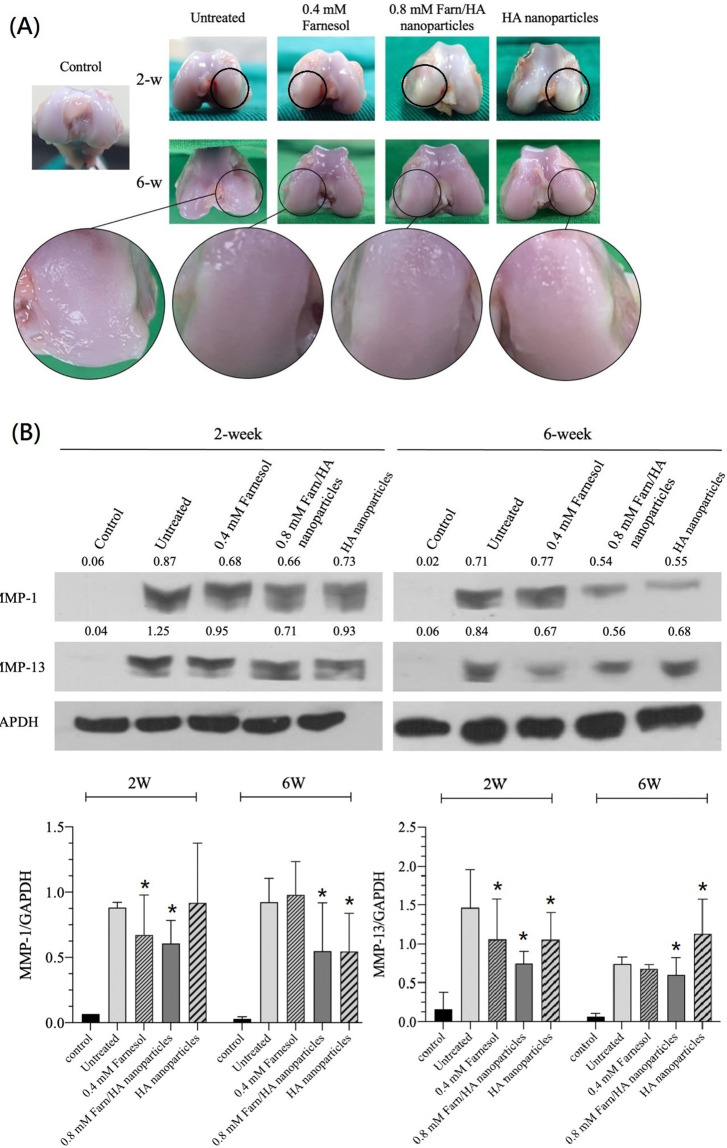
Gross assessment and Western blots test: (a) Gross appearance and assessment of articular cartilage in our surgical resection–induced OA rabbit model after no treatment or treatment with 0.4 mM farnesol, 0.8 mM Farn/HA, or HA nanoparticles after 2 and 6 weeks. Black circles indicate lateral condylar lesions; magnified images of the lesion sites are presented below each image. (b)Western blots for MMP-1 and MMP-13 vs GAPDH in the synovium. *P < 0.05 compared to untreated group.

After 2 weeks, macroscopically, the treated cartilage appeared glossy white and transparent with mostly well-integrated surfaces [Fig. 2(a)]. Clear depressions and scrapes were noted on the untreated lateral condylar cartilage surfaces; by contrast, only a slight color change, without any depressions or wear lesions, was noted on the treated lateral condylar cartilage surfaces. After 6 weeks, the transparency and brightness of the treated cartilage increased. However, the untreated lateral condyle exhibited wear, which caused flattening. The lateral condylar cartilages of the rabbits treated with 0.4 mM farnesol or 0.8 mM Farn/HA exhibited relatively small areas of wear. Notably, the lateral condylar cartilage surfaces treated with 0.8 mM Farn/HA exhibited a rounder surface and thus more repair than did those treated with 0.4 mM farnesol. The cartilage treated with HA nanoparticles exhibited less wear than those in the untreated group but more pronounced wear than did those treated with farnesol. Therefore, on the basis of our observation of gross appearance, treatment with 0.4 mM farnesol, 0.8 mM Farn/HA, or HA nanoparticles leads to reparative effects in lateral condylar cartilage.

### MMP-1 and MMP-13 in the synovium

MMPs, including MMP-1 and MMP-13, are inflammatory factors expressed in OA-affected joint tissues.^24^ A study indicated that both chondrocytes and synovial cells in a rabbit anterior cruciate ligament (ACL) transection model expressed MMP-1; this expression was correlated with cartilage degradation.^25^ Therefore, synovitis may be indicative of cartilage tissue breakdown or degradation.

We harvested the synovium behind the patellar tendon after 2 and 6 weeks of treatment. Total proteins were isolated from the synovium, and MMP-1 and MMP-13 levels were analyzed through Western blotting. At two weeks following treatment, MMP-1 and MMP-13 levels were higher in the untreated cartilage than in the OA-affected cartilage treated with 0.4 mM farnesol, 0.8 mM Farn/HA, or HA nanoparticles and in the normal (control) cartilage [Fig. 2(b)]. MMP-1 and MMP-13 levels decreased with prolongation of the treatment period to 6 weeks—demonstrating the anti-inflammatory effects of farnesol and HA. Treatment with 0.8 mM Farn/HA led to the lowest MMP-1 and MMP-13 levels.

MMP-13 is an indicator of initial joint degeneration and degradation as well as OA progression. Among all the treatment groups, 0.8 mM Farn/HA led to the largest decrease in MMP-13 level, which may be attributable to the slow release of encapsulated farnesol, prolonging the effects compared with 0.4 mM farnesol alone. HA nanoparticles also exerted an anti-inflammatory effect on OA-affected cartilage.

### Histological and histochemical evaluation of lateral femoral condylar cartilage

**FIG. 3.**
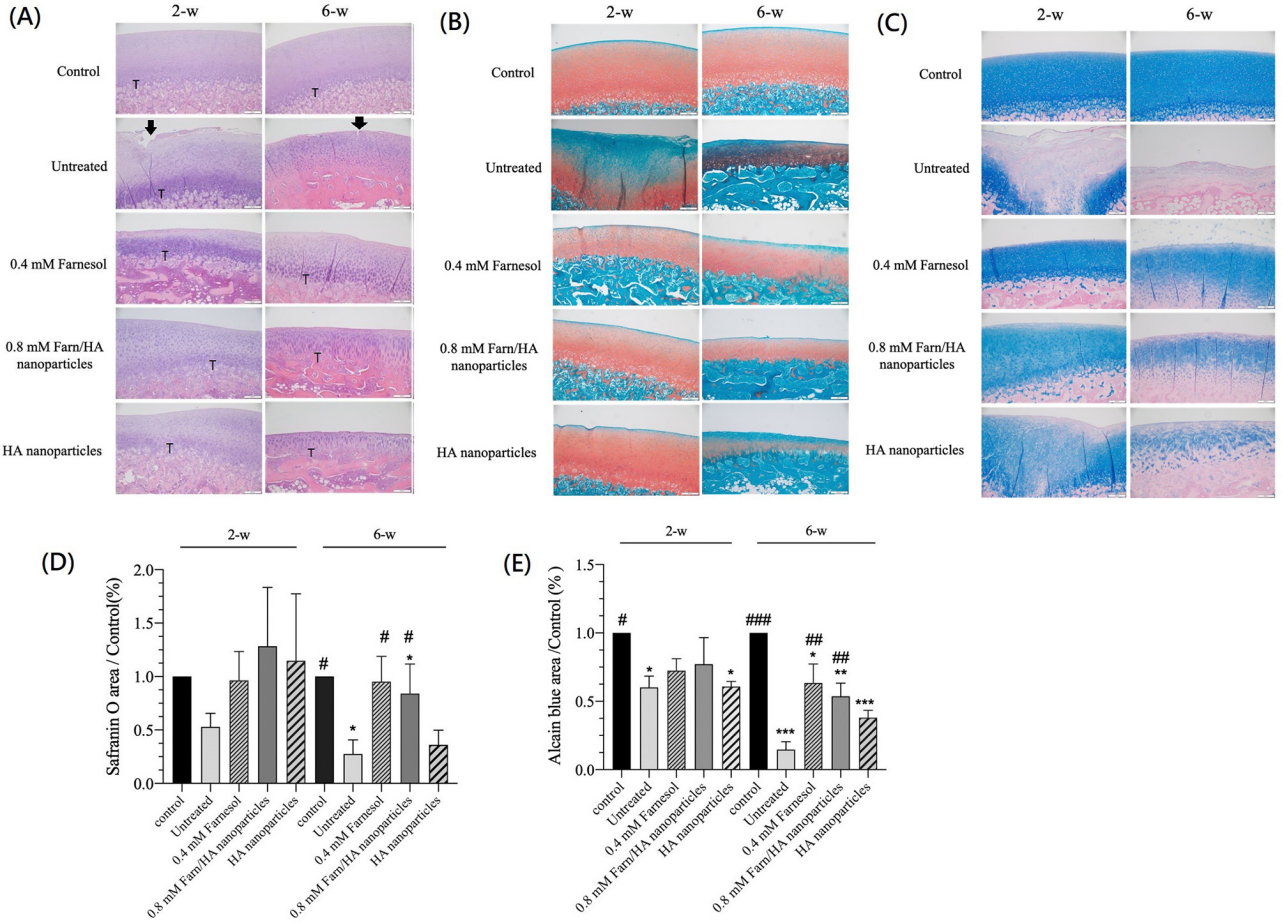
Stains of sections of the articular cartilage of the lateral condyle: (a) H&E: tidemark labeled by T, no tidemark was visible in 6 weeks-untreated group; and focal fibrillation labeled by the black arrow was seen in the untreated group, (b) Safranin O, and (c) Alcian blue after 2 and 6 weeks of treatment. Semiquantitative analysis of (d) Safranin O and (e) Alcian blue staining between groups. Magnification ×100. *p < 0.05, **p < 0.01, and ***p < 0.001 as compared with the control group. #p < 0.05, ##p < 0.01, and ###p < 0.001 as compared with the untreated group (n = 3).

Figure 3(a) presents the H&E-stained sections of lateral condylar cartilage after 2 and 6 weeks of treatment. The normal (control) cartilage tissue exhibited a smooth surface, with the surrounding matrix and associated chondrocytes appropriately oriented in three well-defined zones but without any enlargement or distortion in the chondrons or any proliferative activity in the chondrocytes. In the untreated OA-affected cartilage, the surface had prominent clefts and depressions; its focal fibrillation extended into the midzone portion through the superficial zone, and matrix loss occurred in the midzone with cell death. Moreover, no tidemark was visible in the areas due to deep wear. The cartilage treated with 0.4 mM farnesol or 0.8 mM Farn/HA for 2 weeks exhibited a relatively smooth surface with an intact superficial zone and no apparent fibrillation. Compared with 0.8 mM Farn/HA, 0.4 mM farnesol led to significantly more hypertrophic and clustered chondrocytes, which occupied a large area of the cartilage layer. The cartilage treated with HA nanoparticles, although generally smooth in appearance, exhibited depressions on the surface, all concentrated and pronounced in one area. Also, the tidemark was not clearly visible, and the chondrocytes had shrunken and appeared disoriented, and their number had also decreased. After 6 weeks of treatment, the surface of the untreated OA-affected cartilage had pronounced multiple fibrillation through the superficial zone. By contrast, no fibrillation was noted on the surface of the cartilage treated with 0.4 mM farnesol. However, the chondrocytes in the deeper zone displayed significant hypertrophy, and the chondrocytes appeared sparse in the superficial zone. Compared with the other groups, OA-affected cartilage treated with 0.8 mM Farn/HA had a smoother surface, the entire layer of cartilage is filled with chondrocytes, which are denser than other treatments and have slight hypertrophy. In the cartilage treated with HA nanoparticles, the surface was generally smooth with some frayed areas. The tidemark was not clearly visible, and the subchondral bone had undergone degenerative changes. The chondrocytes appeared to be disorganized, unevenly distributed, and few in number.

Safranin O staining is a cationic indicator dye, which can stain acidic proteoglycans within cartilage. After 2 weeks of treatment, Safranin O staining of the untreated cartilage revealed heterogeneity in the cartilage matrix and proteoglycan depletion in the lower two-thirds of the cartilage layer. One loading with fissure breaks in the untreated group indicated the cartilage matrix defect. The cartilage treated with HA nanoparticles exhibited similar mild heterogeneity and depletion of the cartilage matrix. By contrast, the cartilage treated with 0.4 mM farnesol or 0.8 mM Farn/HA did not exhibit obvious cartilage matrix loss [Fig. 3(b)]. After 6 weeks of treatment, similar cartilage matrix degradation was observed in the untreated and HA nanoparticle-treated cartilage; nevertheless, the cartilage treated with HA nanoparticles demonstrated less matrix degradation than did the untreated cartilage. By contrast, treatment with 0.4 mM farnesol or 0.8 mM Farn/HA enabled OA-affected cartilage repair and intact cartilage matrix retention.

We next used Alcian blue staining to evaluate GAG content in the repaired tissue compared with the surrounding host cartilage. GAG content was relatively high in the cartilage treated with farnesol for 2 and 6 weeks [Fig. 3(c)]. On the surface of the untreated and HA nanoparticle-treated cartilage, most of the repaired tissue exhibited fibrocartilage formation and weak blue staining. After 2 and 6 weeks of treatment with 0.4 mM farnesol and 0.8 mM Farn/HA, blue staining of the cartilage surface became stronger, demonstrating the reparative effect of farnesol in the OA-affected cartilage. These histological and histochemical results indicate that 0.4 mM farnesol and 0.8 mM Farn/HA can restore the chondrocyte phenotype and repair OA-affected cartilage.

As illustrated in Figs. 3(d) and 3(e), we determined Safranin O and Alcian blue staining intensity after 2 and 6 weeks of treatment semiquantitatively. The untreated cartilage demonstrated the lowest Safranin O staining intensity, revealing extensive degradation of the cartilage matrix. The degree of degeneration in the untreated group after 6 weeks of treatment was higher than that after 2 weeks of treatment. By contrast, the cartilage treated with 0.4 mM farnesol, 0.8 mM Farn/HA, and HA nanoparticles exhibited relatively high Safranin O staining intensity, indicating that the cartilage was repaired. Among all treatment groups, HA nanoparticles led to the least cartilage matrix reparative effect. The results for Alcian blue staining were consistent with those for Safranin O staining. Taken together, these findings demonstrate that 0.4 mM farnesol and 0.8 mM Farn/HA have considerable reparative effects in OA-affected cartilage.

### IHC for MMP-13 in lateral femoral condylar cartilage

**FIG. 4.**
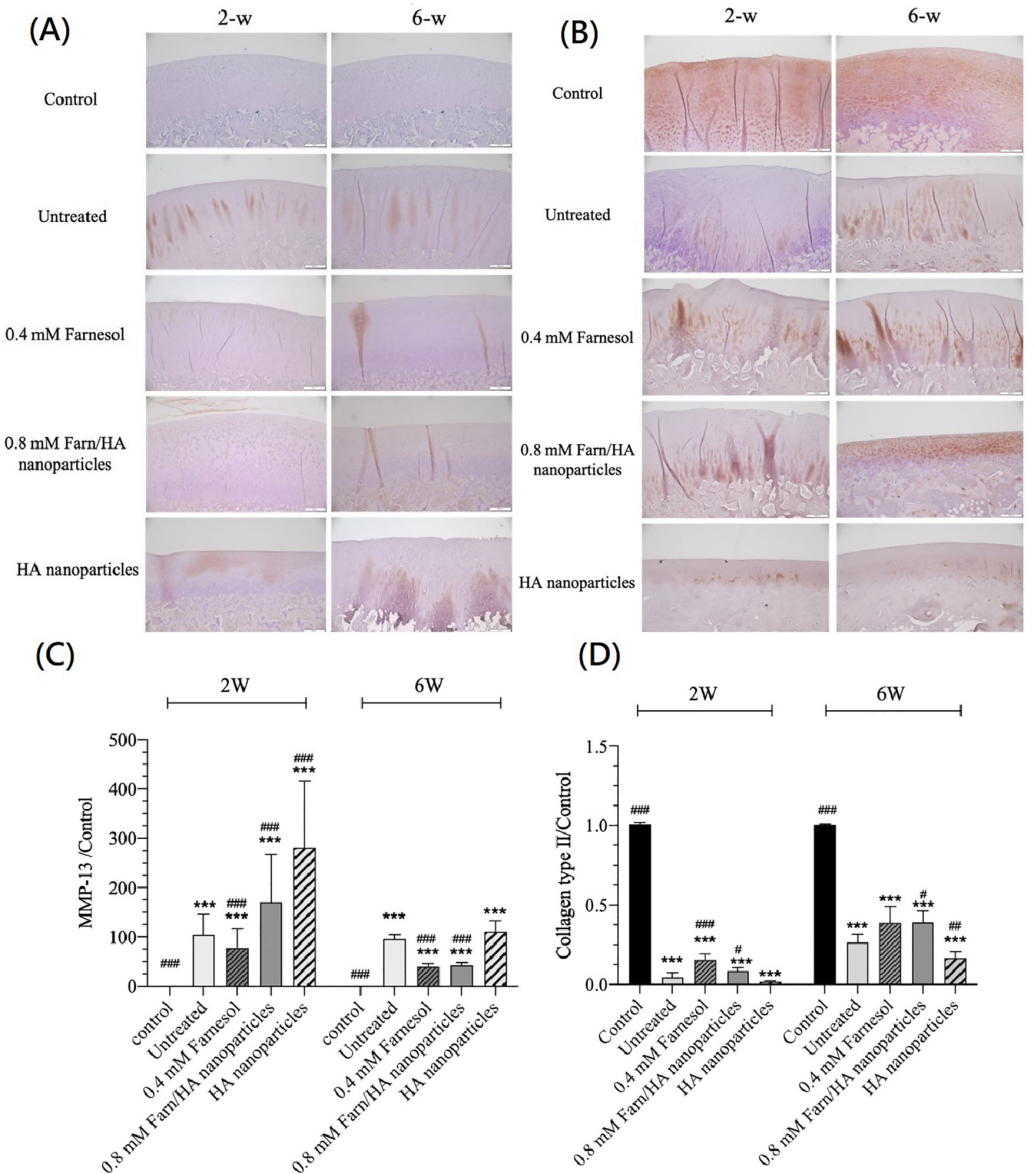
IHC stain and Semiquantitative analysis: (a) IHC for MMP-13 in lateral condylar cartilage (brown) after 2 and 6 weeks of treatment. Magnification ×100. (b) IHC for COL II of lateral condylar cartilage with OA (brown) after 2 and 6 weeks of treatment. Semiquantitative analysis of (c) MMP-13 and (d) COL II staining between groups. Magnification ×100. ***p < 0.001 as compared with the control group. #p < 0.05, ##p < 0.01, and ###p < 0.001 as compared with the untreated group (n = 3).

MMP-13 has a major role in the pathology of early OA because it can initiate the degradation of a wide range of downstream matrix and collagen components. Here, we detected the presence of MMP-13 in the harvested lateral femoral condylar cartilage through IHC. The untreated cartilage had an intense brown color dispersed throughout the cartilage layer, indicating the presence of MMP-13 throughout the cartilage [Fig. 4(a)]. All cartilage treated with farnesol in any form, particularly 0.8 mM Farn/HA, exhibited lighter or smaller brown areas. While the brown staining in the HA group was not as extensive as in the untreated group, significant staining was still observed in specific areas as compared to the farnesol group. These results indicate that the untreated cartilage had higher MMP-13 levels, indicating more OA-related degeneration and degradation. However, after 2 weeks of treatment with 0.4 mM farnesol or 0.8 mM Farn/HA, the levels of MMP-13 decreased, suggesting that farnesol effectively protects cartilage tissue from degradation.

### IHC for COL II

We next used IHC to detect COL II production after treatment. A more intense brown color, indicating the presence of higher levels of COL II, observed in the cartilage treated with 0.4 mM farnesol or 0.8 mM Farn/HA than in the untreated and HA nanoparticle-treated cartilage [Fig. 4(b)]. The cartilage treated with 0.8 mM Farn/HA displayed stronger staining with more even distribution throughout than did the cartilage treated with 0.4 mM farnesol. By contrast, in the untreated and HA nanoparticle-treated cartilage, COL II production was sporadic. Therefore, farnesol improved COL II production in OA-affected cartilage, facilitating its repair. Among of them, treatment with 0.8 mM Farn/HA is more effective than 0.4 mM farnesol.

As illustrated in Figs. 4(c) and 4(d), we semiquantitatively determined IHC MMP-13 and COL II staining intensity after 2 and 6 weeks of treatment. The control (normal) groups demonstrated the lowest MMP-13 and highest COL II staining intensity, revealing health cartilage. The untreated group exhibited mild inflammation after 6 weeks of treatment. The inflammation reaction was alleviated after 6 weeks of treatment with 0.4 mM farnesol, 0.8 mM Farn/HA exhibited relatively low MMP-13 staining intensity, indicating that farnesol exerted an anti-inflammatory activity on the cartilage. The results for COL II staining demonstrate that 0.4 mM farnesol and 0.8 mM Farn/HA have considerable capability to produce COL II in OA-affected cartilage after 6 weeks of treatment. However, a longer period of 6 weeks of treatment is needed to acquire the anti-inflammation response and COL II production to repair the OA-affected cartilage.

### OA grading and staging

We used OARSI's system to evaluate OA progression on the basis of the histological features of our cartilage samples; it aided in understanding the severity of cartilage damage and the repair efficacy of the treatment with OA progression. The untreated cartilage, with a combined score of 5, demonstrated considerable deterioration after 2 weeks (Table I). After 6 weeks, the combined score increased to 9, indicating the progression of OA. By contrast, the cartilage treated with 0.4 mM farnesol or 0.8 mM Farn/HA exhibited much lower scores (1–3), with a more intact cartilage matrix and healthy cartilage surface after 2 and 6 weeks of treatment, indicating the reparative effect of farnesol. However, the HA nanoparticle-treated group exhibited cartilage degeneration and matrix loss, yielding a combined score of 5, after 6 weeks of treatment.

**TABLE I.** Analysis based on OARSI's system.

	Grade	Stage	Score
	(0–6.5)	(0–4)	(Grade × stage)
Groups (2-w after treatment)			
Control	0	0	0
Untreated	2.5	2	5
0.4 mM Farnesol	1.5	1	1.5
0.8 mM Farn/HA nanoparticles	1.0	1	1
HA nanoparticles	2.0	1	2
Groups (6-w after treatment)			
Control	0	0	0
Untreated	3	3	9
0.4 mM Farnesol	1.5	2	3
0.8 mM Farn/HA nanoparticles	1.5	2	3
HA nanoparticles	2.5	2	5

## DISCUSSION

Articular cartilage is a highly complex tissue that can withstand tremendous force over a long period but cannot heal itself, even after a minor injury. Arthritis is caused by the absence of articular cartilage in a joint; joint health and function are dependent on cartilage viability. Chondrocytes are formed from mesenchymal stem cells and account for approximately 2% of the total volume of articular cartilage. They are highly specialized, metabolically active cells that play a unique role in ECM formation, maintenance, and repair.^26^ Chondrocytes rarely form cell–cell contact that transmit signals and communicate directly with each other. However, they respond to various stimuli, including those from growth factors, mechanical loads, piezoelectric forces, and hydrostatic pressures.^5^ Their synthetic activity increases during the early stages as they attempt to repair the damage. However, after disruption of their pericellular matrix, chondrocytes become exposed to factors and components that influence their phenotype and behavior. After the cartilage matrix is weakened by proteolytic activity, even non-injurious loads may be perceived as injurious by the chondrocytes, resulting in a cycle of chronic, mechanically induced matrix breakdown.

Articular cartilage has a structure similar to the cartilage of the growth plate. Patients with OA and several animal OA models have been observed to exhibit numerous pathological features typically associated with endochondral ossification of the growth plates, and these features have been associated with disease severity.^27,28^ In addition to chondrocyte proliferation and hypertrophy, superficial hyaline cartilage thinning, calcium phosphate crystal deposition, osteochondral remodeling, and cartilage calcification,^29^ increased expression of ossification-related proteins such as runt-related transcription factor 2, MMP-13, osteopontin, vascular endothelial growth factor, and osteocalcin is observed during skeletogenesis; these proteins are silenced in adult articular cartilage but re-expressed during OA. Inhibiting articular chondrocyte proliferation, hypertrophy, vascular erosion, subchondral bone remodeling, and other key events in the process of endochondral ossification may enable effective OA treatment.

Transcription factor SOX9 is essential for embryonic chondrogenesis. Jacques *et al.* reported that SOX9 is required postnatally to prevent growth plate closure and preosteoarthritic cartilage deterioration.^30^ SOX9 upregulation may suppress a disintegrant and metalloproteinase with thrombospondin motifs (ADAMTS) in the early stage of OA in humans. Zhang *et al.* assessed whether SOX9 mediates ADAMTS dysregulation during cartilage degeneration^31^ and reported that SOX9 expression was negatively correlated with ADAMTS production in 22 randomly selected patients with OA. In addition, the inflammatory cytokines TNF-α and IL-1β repress SOX9 activity and activate ADAMTS expression, promoting OA in humans during its early stages.

In load-bearing areas, SOX9 deficiency can result in chondrocyte-to-osteoblast conversion during the progenitor stage. In this phase of cell lineage transition, SOX9 may play a role in controlling the activation of transforming growth factor β (TGF-β) and bone morphogenetic protein (BMP) signaling pathways.^30^ The TGF-β family of polypeptide growth factors control the development and homeostasis of many tissues, including articular cartilage. The TGF-β family has more than 30 members, including TGF-β, activins, BMPs, and growth-differentiation factors.^32^ Each TGF-β family member plays a specific role in cartilage biology and OA development, including inflammation, ECM production and degradation, and chondrocyte proliferation and hypertrophy.^32^ Mutual regulation between TGF and SOX9 is an essential part of ECM protein production. TGF-β regulates the phosphorylation and stabilization of SOX9 in chondrocytes through p38 and Smad-dependent mechanisms.^33^ In addition, SOX9 is required for regulation of *PAPSS2* mRNA expression by TGF-β; PAPSS2 is an enzyme essential for proteoglycan sulfation.^34^

Chondrocytes are generally isolated from articular cartilage through collagenase reaction and proliferated through *in vitro* monolayer culture. However, after multiple passages, the phenotype, including morphology and matrix, of chondrocytes alters; this is referred to as dedifferentiation.^35^ In dedifferentiated chondrocytes, the phenotype gradually changes from polygonal to fibroblastic, and the secreted collagen type changes from COL II to type I collagen and COL X; their proteoglycan secretion and chondrocyte proliferation rates also decrease. Dedifferentiation also refers to the loss of function and characteristics. During the dedifferentiation process, the signal from the Indian hedgehog protein (IHH) induces chondrocyte hypertrophy and upregulates COL X and MMP expression in OA chondrocytes. Thus, increased IHH expression is associated with OA severity and chondrocyte hypertrophy markers expression; the markers include COL X, MMP-13, and chondrocyte size.^36^ IL-1β can stimulate nuclear factor kB, which regulates nitric oxide (NO) and prostaglandin E2 (PGE2) production, thus activating MMP-1 and MMP-13 secretion and modulating inflammation, ECM synthesis, and apoptosis.^37^ In addition, IL-1β inhibits proteoglycan and collagen synthesis. We previously investigated the dedifferentiation of IL-1β-stimulated chondrocytes in response to farnesol treatment by measuring MMP-1, inducible NO synthase (iNOS), and IL-6. We concluded that levels of these proteins were higher in the IL-1β-stimulated chondrocytes than in the normal chondrocytes. After treatment with farnesol, these dedifferentiation- and inflammation-related proteins decreased the IL-1β-stimulated chondrocytes to levels similar to those in normal chondrocytes.^22^

In the present study, the untreated OA-affected cartilage had higher MMP-1 and MMP-13 levels after 2 weeks; these levels increased further after 6 weeks, indicating OA progression [Fig. 2(b)]. By contrast, treatment with 0.4 mM farnesol or 0.8 mM Farn/HA reduced MMP-1 and MMP-13 levels. These levels continued to decrease with the progression of the treatment, indicating the anti-inflammatory effects of farnesol, which inhibited inflammation and reduced degeneration in the OA-affected cartilage. HA nanoparticles not only demonstrated considerable anti-inflammatory effects but also reduced MMP levels. However, the anti-inflammatory effect of HA was weaker than that of farnesol. In addition, 0.8 mM Farn/HA exerted more prolonged anti-inflammatory effects than did 0.4 mM farnesol, potentially because Farn/HA contained HA and enabled the slow and sustained release of farnesol.

Our gross morphology observations revealed clear and extensive wear in the untreated lateral condyle. After treatment with 0.4 mM farnesol or 0.8 mM Farn/HA, the OA-affected cartilage exhibited minor wear [Fig. 2(a)]. We previously noted that farnesol restores the production of chondrogenic COL II and suppress the production of COL I in IL-1β-stimulated dedifferentiated chondrocytes.^22^ In addition, treatment with farnesol significantly reduces PGE2 secretion to a level similar to that secreted the normal chondrocytes. PGE2, involved in inflammation and OA symptom development, modulates PGE2-derived signaling and influences chondrocyte metabolism, affecting ECM structure. The decrease in PGE2 levels indicates that farnesol suppresses inflammatory responses in IL-1β-stimulated chondrocytes, thereby likely contributing to the restoration of chondrocyte function.^38^ Alteration in GAG is associated with articular cartilage degeneration and maintenance of appropriate ECM component concentrations. Wu *et al.* also indicated that GAG production resumes and increases in the presence of 0.4 mM farnesol or 0.8 mM Farn/HA.^22^ In the present *in vivo* study, H&E staining revealed that 0.8 mM Farn/HA led to a greater increase in the number of chondrocytes without significant hypertrophy than did 0.4 mM farnesol or HA nanoparticles [Fig. 3(a)]. Therefore, 0.8 mM Farn/HA can retain or restore chondrocyte phenotypes in the OA-affected cartilage and repair the defect thereafter. COL II in the ECM is a phenotype of healthy cartilage. According to our results of IHC for COL II, 0.8 mM Farn/HA had higher COL II production than did 0.4 mM farnesol or HA nanoparticles [Fig. 4(b)]. This demonstrates the benefits of encapsulating farnesol in HA, which enables the sustained release of farnesol in the OA-affected cartilage.

HA, an essential ECM component, binds to ECM molecules and receptors on cell surfaces to control cell behaviors such as proliferation, migration, development, recognition, and morphogenesis as well as some physiological functions. Because HA has unique properties and is a constituent of GAG and articular cartilage, intraarticular HA injection may benefit the joints by improving joint lubrication. In addition, HA can exert a cellular effect in dedifferentiated chondrocytes.^39^ HA targeting IL-1β-stimulated chondrocytes has been reported to reduce PGE-2 and COL I but increase COL II levels. However, this effect was not as significant as that of 0.4 mM farnesol or 0.8 mM Farn/HA.^22^ In the present study, histological sections exhibited a sparse chondrocyte distribution after 6 weeks of farnesol treatment. The chondrocytes appeared to have lost much of their surrounding cartilage matrix.

With the advancement of nanotechnology, the development of nano-based drug delivery systems has become a critical research direction. The size of nanoparticle carriers ranges from 1 to 100 nm. They are widely used in various biomedical fields to improve the solubility, reduce the side effects or toxicity, and increase the stability and efficiency of prominent drug molecules in the target tissues.^40^ Our laboratory has established an electrostatic field system to prepare nanoparticles for clinical use and encapsulate cancer treatment drugs.^41^ In this study, we used this system to produce our HA nanoparticles in an aqueous environment to deliver farnesol and increase biocompatibility, all with satisfactory homogeneity and uniform size.^22^ After 24 h of incubation with <0.4 mM farnesol, chondrocyte viability did not decrease significantly; the IC_50_ of farnesol and Farn/HA was approximately 0.5 and 1.9 mM, respectively. Encapsulating compounds and drugs in HA nanoparticles can reduce the inhibitory effects of drugs on cell viability; in addition, encapsulating a relatively large proportion of the drug in HA nanoparticles may enhance the drug's therapeutic effects through slow drug release.^41^ Therefore, we selected 0.4 mM farnesol and 0.8 mM Farn/HA to assess the effects of farnesol treatment.

Adding HA to biomaterial scaffolds can significantly accelerate SOX9 and COL II expression in the early stage of the cell cycle.^42,43^ Wu *et al.* used HA as a carrier material to encapsulate farnesol.^22^ The authors observed that Farn/HA exerted superior anti-inflammatory and restoration effects over a long-term culture period; this effect is attributable to the slow release of farnesol.^41^ In the current animal study, we investigated the initial inflammatory signal on the basis of the synovial MMP levels and observed that 0.8 mM Farn/HA can reduce MMP levels as quickly as 0.4 mM farnesol. However, the effects of 0.4 mM farnesol were not sustained in the long term (i.e., for 6 weeks).

Treatment of joint degeneration remains challenging, mainly because cartilage degeneration is almost irreversible after it starts. Various surgical procedures have been developed that have improved numerous patients' quality of life; however, specific postsurgical complications, or even nonresolution of the original symptoms, have been noted in some patients. Several crucial processes after cartilage injury or degeneration can be addressed using farnesol because of its antioxidant, anti-inflammatory, and collagen production-promoting effects.^44^ According to our results, early intervention can aid in combatting inflammation and preventing subsequent cartilage damage. Adding farnesol to cultured chondrocytes can reduce PGE2 and iNOS production; in addition, increased SOX9 production can prevent degradation in the ECM.^22^

In summary, our *in vivo* animal study demonstrated the following benefits of farnesol, specifically Farn/HA, in cartilage:

1. It can reduce superficial cartilage damage caused by MMPs and other inflammatory phenomena.
2. It can prevent hypertrophy of chondrocytes with loss of function and even restore the functional chondrocyte phenotype.
3. It can increase COL II and GAG production, enhancing repair and damage prevention ability of the cartilage; it can also aid in retaining a large amount of COL II and GAG in the cartilage matrix.

Additional studies with a comprehensive experimental design on the long-term effects of farnesol for severe cartilage injury prevention and post-superficial injury cartilage restoration is warranted.

Limitations of the study included the small sample size and short follow-up time. A larger sample size and longer follow-up may be required to study the progression of cartilage repair in the future.

## Conclusion

Farnesol and Farn/HA reduced inflammatory responses by decreasing the levels of inflammatory factors MMP-1 and MMP-13 in rabbits with surgical resection-induced OA. Reducing MMP levels at the initial stage of injury might delay or prevent subsequent cartilage matrix degradation. Farnesol was noted to restore the chondrocyte phenotype and improve the COL II production. In addition, farnesol significantly increased GAG secretion in the OA-affected cartilage, aiding its repair. The use of Farn/HA may enable farnesol-based therapy at relatively safe and effective concentrations, and the slow release of farnesol from Farn/HA may facilitate relatively long-term cartilage protection. In general, farnesol exerts preventative and reparative effects on cartilage affected by OA.

## METHODS

### Materials

Farnesol, hyaluronan (HA, MW: 9 × 10^5^ Da), 3–4,5-dimethylthiazol-2-yl-2,5-diphenyltetrazolium bromide (MTT), and FeCl_3_ were purchased from Sigma (St. Louis, MO, USA). F12 medium, fetal bovine serum, streptomycin, and penicillin were obtained from Gibco (Waltham, MA, USA). All other chemicals used in this study were of analytical reagent grade. Animal experiments conducted in this study were approved by the Institutional Animal Care and Use Committee of I-Shou University, Kaohsiung, Taiwan (IACUC-ISU-108–035, approval date: 12 February 2020).

### Farnesol-HA nanoparticle production and characterization

**FIG. 5.**
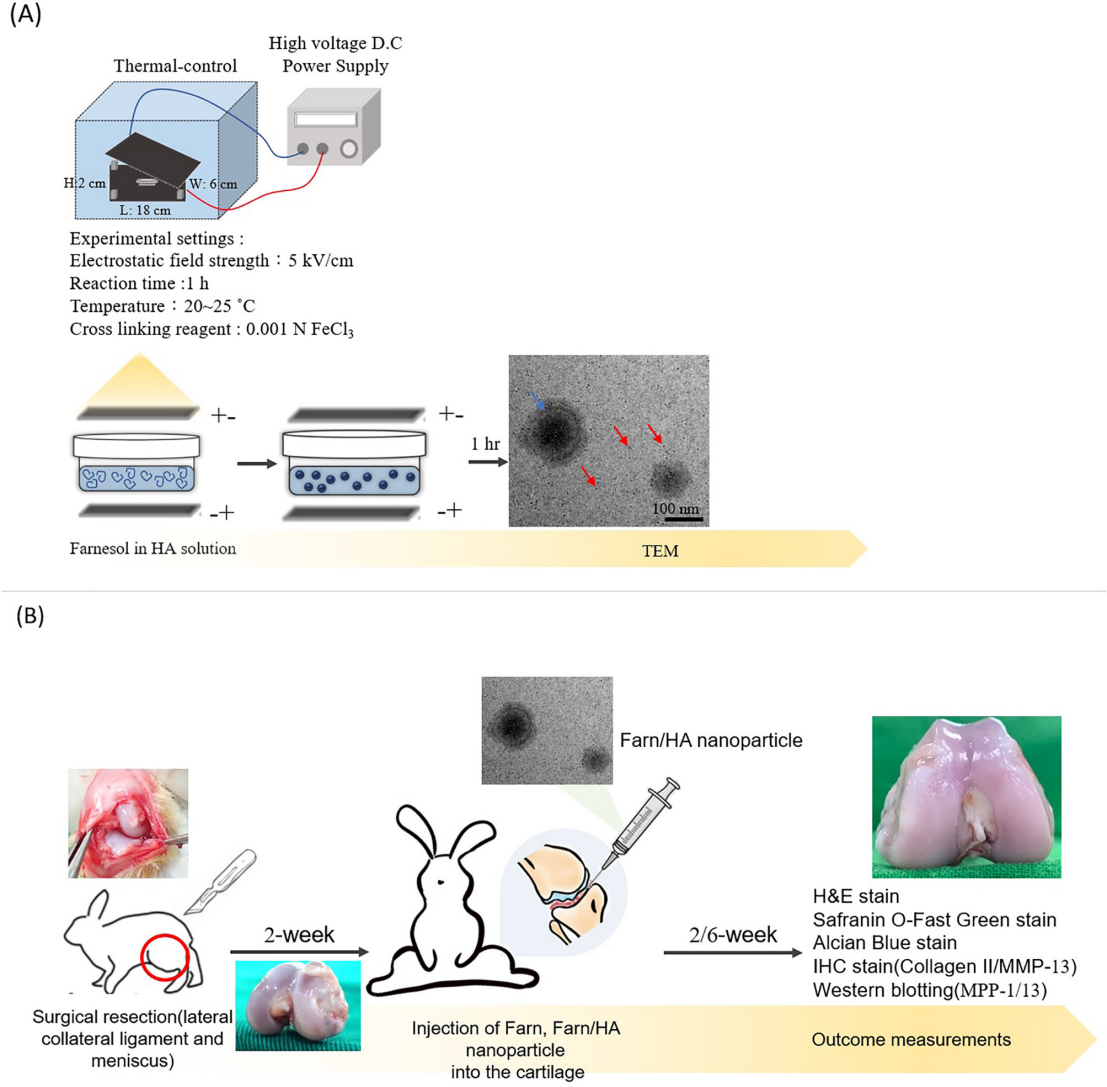
Schematic for study: (a) preparation of HA nanoparticles and Farn/HA. In short, 1 ml HA (0.2 mg/ml) solution was mixed with 30 *μ*l and electrosprayed under the settings with 5 kV/cm for 1 h at 20–25 °C and collected in a cross-linking solution (ferric chloride, 0.001N) to produce Farn/HA nanoparticles. HA nanoparticles were labeled by a red arrow and Farn/HA nanoparticles by a blue arrow. (b) *In vivo* experimental design for the evaluation of farnesol and Farn/HA nanoparticles on the repair and prevention of OA.

HA nanoparticle-encapsulated 0.8 mM farnesol (Farn/HA) was fabricated using an electrostatic field system in accordance with the methods used in other reports.^22^ A stock solution of 1.2 M farnesol was prepared by dissolving farnesol in dimethyl sulfoxide (DMSO). Subsequently, 1 ml of 0.2 mg/ml HA was mixed with 30 μl of farnesol. The mixture was transferred onto a petri dish and placed between two plate electrodes. Our Farn/HA preparation parameters in the electrostatic field system are illustrated in Fig. 5(a). The morphology of the prepared HA nanoparticle, and the Farn/HA nanoparticle was examined using a Tecnai G2 20 S-Twin transmission electron microscope (TEM; FEI, Hillsboro, OR, USA). The size of the HA nanoparticle, and the Farn/HA nanoparticle was estimated from the TEM images by randomly sampling approximately 50 individual nanoparticles using Image J software (Version 1.50; National Institutes of Health, Bethesda, MD, USA). The prepared farnesol-encapsulated nanoparticles were centrifuged at 12 000 rpm for 20 min, while the free farnesol in the supernatant was qualified and quantified through high-performance liquid chromatography (HPLC, Agilent 1100 series, Santa Clara, CA, USA). The encapsulation efficiency (EE%) of the farnesol was calculated using the following formula: EE (%) = (AFa/AFb) × 100%, where AFa is the amount of farnesol in the nanoparticle found after centrifugation. AFb is the amount of farnesol in nanoparticles found before centrifugation. The *in vitro* farnesol release rate was measured by calculating the free farnesol in the supernatant at each predetermined time. *In vitro* release (%) = [(total amount of farnesol-residue of farnesol)/total amount of farnesol] ×100%.^20^

### Cell viability

The effects of farnesol and Farn/HA on chondrocyte viability were assessed using the MTT assay. The chondrocytes (passage 2) were seeded onto 96-well plates at 7 × 10^3^ cells/well for 24 h and subsequently treated with farnesol or Farn/HA at various concentrations in triplicate. The absorbance was measured at 570 nm using a multiplate reader (Thermo Scientific, Waltham, MA, USA).

### Assessment of OA repair in vivo

#### Experimental design and animal model

OA was induced in the cartilage of 8-week-old male New Zealand rabbits through resection of the lateral collateral ligament and meniscus. All the animals were obtained 1 week before the experiment and raised in the same environment. Each rabbit was housed in its individually ventilated cage with filter lids, provided with sterilized bedding and environmental enrichment, maintained at 21–22 °C with a 12-h light/dark cycle and water and complete pelleted food.

**FIG. 6.**
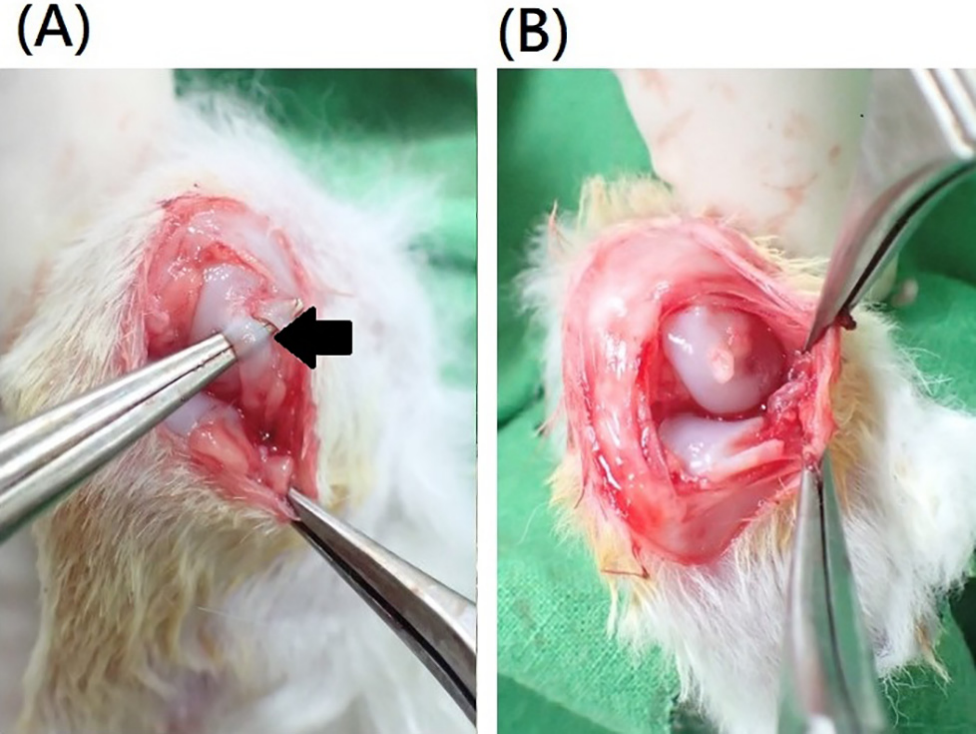
Animal model of OA: (a) arthrotomy of rabbit knee joint, before resection of lateral collateral ligament (black arrow) and (b) after lateral collateral ligament resection and lateral meniscectomy.

The right stifle was prepared in a surgically sterile manner. A midline incision of approximately 3 cm was created under knee flexion. The patella was medially dislocated using lateral parapatellar arthrotomy to achieve optimal visualization of the lateral collateral ligament and meniscus. The intraarticular structures were then thoroughly examined to detect any abnormalities, such as infection or deformity. Both the total lateral meniscectomy and lateral collateral ligament resection were performed to induce subsequent cartilage injury and degeneration (Fig. 6).^23^ The clinical signs of pain, salivation, health status, and abnormal behavior were carefully monitored during procedures.

A simple randomization method with an excel file was used to allocate experimental units to control and treatment groups. The rabbits were randomized into a control group (n = 2 healthy rabbits) and four experimental groups (n = 6 in each group): untreated rabbits with OA, OA rabbits who received 0.4 mM farnesol, OA rabbits who received 0.8 mM Farn/HA, and OA rabbits who received pure HA nanoparticles. N = 6 in each experimental group was designed for statistical significance. The animals were kept in cages until recovery from anesthesia; subsequently, the animals were allowed to move freely for 2 weeks. During the experiment, rabbits were excluded if their health status became ill or died.

Next, the diseased limb was treated with intraarticular injection of 0.5 ml of 0.4 mM Farnesol, 0.8 mM Farn/HA, or pure HA nanoparticles [Fig. 5(b)].

#### Histopathological analysis

After 2 and 6 weeks of treatment, the rabbits were sacrificed; next, a midline incision was made to expose the patella, muscles, and patellar tendons. Parapatellar arthrotomy was performed again; this was followed by the separation of the anterior and posterior cruciate ligaments as well as the remaining collateral ligaments and joint capsules. The condyle of the femur was removed such that the cartilage layer was not damaged in the process.

Osteochondral specimens harvested from the lateral femoral condyles were dehydrated in graded ethanol solutions, cleared in xylene, embedded in paraffin blocks, and sliced into 4-mm-thick sagittal sections. The target slabs are the main weight-loading area at the middle third of the lateral femur condyle. Next, these sections were stained using hematoxylin and eosin (H&E) for histological examination.

We used Osteoarthritis Research Society International's (OARSI's) osteoarthritis cartilage histopathology assessment system to assess cartilage pathology and evaluate the histological OA repair and prevention effects of the treatment. This system measures lesion depth in seven grades and divides the extent of OA over a joint surface into five stages. The stages are determined based on the extent of cartilage surface, area, and volume involved in the local OA process—from stage 0 (normal) to stage 4 (>50%). OA is graded from 0 (intact surface) to 6 (complete loss of cartilage and bone deformation). We also considered subgrades—ranging from 1.0 (intact cells) to 6.5 (joint margin and central osteophytes). Next, we calculated joint scores by multiplying the stage with the grade; the lower the combined score was, the more successful was the OA-affected cartilage repair or OA prevention. Two orthopedic surgeons, both experts in arthritis and blinded to the experimental details, conducted the scoring.

#### Histochemical analysis

After the macroscopic observation, the treated and untreated cartilage samples were fixed in 10% buffered formalin, treated with Surgipath Decalcifier (Thermo Scientific, Pittsburg, PA, USA), dehydrated in ascending graded series of ethanol, and sliced into 4-mm-thick sagittal sections. These sections were then stained with H&E for histological evaluation and with Safranin O and Alcian blue for GAG content estimation.

#### Immunohistochemistry

For immunohistochemistry (IHC), the sections were deparaffinized and rehydrated using graded concentrations of ethanol and then de-ionized water. The sections were incubated in a hydrogen peroxide blocking solution for 10 min and then washed with phosphate-buffered saline (PBS). The sections were subjected to heat treatment (at 95 °C) in 0.01 M sodium citrate buffer with Tween 20. Each section was incubated with ImmunoBlock (PBS, pH 7.6, with 0.5% bovine serum albumin and <0.1% sodium azide) at room temperature for 20 min, followed by washing with PBS. Next, they were incubated with rabbit antimouse COL II at 4 °C overnight and then with Mouse/Rabbit Probe HRP Labeling solution at room temperature for 30 min. Finally, 3,3′-diaminobenzidine was applied for 10 min. The intensity of the brown color, indicating the presence of COL II, was quantified using ImageJ.

#### Western blotting

Total protein was isolated from treated and untreated cartilage samples by using the RIPA buffer, containing a phosphatase and protease inhibitor cocktail. The samples were incubated on ice for 30 min and centrifuged at 13 000 rpm at 4 °C for 15 min. Their protein concentration was determined using a BCA protein assay kit (GeneMark, Atlanta, GA, USA). In total, 30 *μ*g of protein was loaded onto sodium dodecyl sulfate polyacrylamide gels and separated through electrophoresis. Subsequently, the proteins were transferred onto polyvinylidene difluoride membranes (PALL, New York, CA, USA). Next, the membranes were blocked with 5% nonfat milk for 1 h and incubated overnight with primary antibodies against MMP-1 (1:500), MMP-13 (1:500), and GADPH (1:10 000) at 4 °C. Thereafter, the membranes were incubated with enzyme-linked secondary antibodies at room temperature for 1 h. MMP-1 and MMP-13 protein levels were compared between the groups by using a semiquantitative intensity analysis on ImageJ.

### Statistical analysis

All values are expressed as means ± standard errors of the means. Significant differences between the experimental and control groups were tested using one-way analysis of variance. All analyses were performed on IBM SPSS Statistics for Windows (version 22, IBM Corp., Armonk, NY, USA). A *p* value of <0.05 indicated statistical significance.

## SUPPLEMENTARY MATERIAL

See the supplementary material for the calculation of the nanoparticle size and the raw images of Western blot.

## Data Availability

The data that support the findings of this study are available within the article and its supplementary material.

## References

[1] A. M. Bhosale and J. B. Richardson , “ Articular cartilage: Structure, injuries and review of management,” Br. Med. Bull. 87, 77–95 (2008). 10.1093/bmb/ldn025 18676397

[2] C. A. Poole , “ Articular cartilage chondrons: Form, function and failure,” J. Anat. 191(1), 1–13 (1997). 10.1046/j.1469-7580.1997.19110001.x 9279653 PMC1467653

[3] J. Shi , Q. Liang , M. Zuscik *et al.*, “ Distribution and alteration of lymphatic vessels in knee joints of normal and osteoarthritic mice,” Arthritis Rheumatol. 66(3), 657–666 (2014). 10.1002/art.38278 24574226 PMC4074307

[4] F. Guilak , “ Biomechanical factors in osteoarthritis,” Best Pract. Res. Clin. Rheumatol. 25(6), 815–823 (2011). 10.1016/j.berh.2011.11.013 22265263 PMC3266544

[5] J. A. Buckwalter and H. J. Mankin , “ Articular cartilage: Tissue design and chondrocyte-matrix interactions,” Instr Course Lect. 47, 477–486 (1998). 9571449

[6] G. S. Man and G. Mologhianu , “ Osteoarthritis pathogenesis—A complex process that involves the entire joint,” J. Med. Life 7(1), 37–41 (2014). PMC3956093 24653755

[7] H. S. Kyung , “ High tibial osteotomy for medial knee osteoarthritis,” Knee Surg. Relat. Res. 28(4), 253–254 (2016). 10.5792/ksrr.16.253 27894170 PMC5134785

[8] E. C. Rodriguez-Merchan , “ Medial Unicompartmental Osteoarthritis (MUO) of the Knee: Unicompartmental Knee Replacement (UKR) or Total Knee Replacement (TKR),” Arch. Bone Jt. Surg. 2(3), 137–140 (2014). 25386571 PMC4225015

[9] M. E. Steinhaus , A. B. Christ , and M. B. Cross , “ Total knee arthroplasty for knee osteoarthritis: Support for a foregone conclusion?,” HSS J. 13(2), 207–210 (2017). 10.1007/s11420-017-9558-4 28690473 PMC5481268

[10] Y. Gao , S. Liu , J. Huang *et al.*, “ The ECM-cell interaction of cartilage extracellular matrix on chondrocytes,” Biomed. Res. Int. 2014, 648459. 10.1155/2014/648459 24959581 PMC4052144

[11] P. Singh , K. B. Marcu , M. B. Goldring , and M. Otero , “ Phenotypic instability of chondrocytes in osteoarthritis: On a path to hypertrophy,” Ann. N. Y. Acad. Sci. 1442(1), 17–34 (2019). 10.1111/nyas.13930 30008181

[12] A. C. Hall , “ The role of chondrocyte morphology and volume in controlling phenotype-implications for osteoarthritis, cartilage repair, and cartilage engineering,” Curr. Rheumatol. Rep. 21(8), 38 (2019). 10.1007/s11926-019-0837-6 31203465 PMC6571082

[13] M. Brittberg , A. Lindahl , A. Nilsson , C. Ohlsson , O. Isaksson , and L. Peterson , “ Treatment of deep cartilage defects in the knee with autologous chondrocyte transplantation,” N. Engl. J. Med. 331(14), 889–895 (1994). 10.1056/NEJM199410063311401 8078550

[14] A. G. McNickle , D. R. L'Heureux , A. B. Yanke , and B. J. Cole , “ Outcomes of autologous chondrocyte implantation in a diverse patient population,” Am. J. Sports Med. 37(7), 1344–1350 (2009). 10.1177/0363546509332258 19286911

[15] K. Y. Hsiao , Y. J. Wu , Z. N. Liu , C. W. Chuang , H. H. Huang , and S. M. Kuo , “ Anticancer effects of sinulariolide-conjugated hyaluronan nanoparticles on lung adenocarcinoma cells,” Molecules 21(3), 297 (2016). 10.3390/molecules21030297 26950100 PMC6274027

[16] R. Khan and S. Sultana , “ Farnesol attenuates 1,2-dimethylhydrazine induced oxidative stress, inflammation and apoptotic responses in the colon of Wistar rats,” Chem.-Biol. Interact. 192(3), 193–200 (2011). 10.1016/j.cbi.2011.03.009 21453689

[17] Y. Y. Jung , S. T. Hwang , G. Sethi , L. Fan , F. Arfuso , and K. S. Ahn , “ Potential anti-inflammatory and anti-cancer properties of farnesol,” Molecules 23(11), 2827 (2018). 10.3390/molecules23112827 30384444 PMC6278318

[18] N. Cerca , F. Gomes , J. C. Bento *et al.*, “ Farnesol induces cell detachment from established S. epidermidis biofilms,” J Antibiot. 66(5), 255–258 (2013). 10.1038/ja.2013.11 23549353

[19] C. M. Ku and J. Y. Lin , “ Farnesol, a sesquiterpene alcohol in herbal plants, exerts anti-inflammatory and antiallergic effects on ovalbumin-sensitized and -challenged asthmatic mice,” Evidence Based Complementary Alternat. Med. 2015, 387357. 10.1155/2015/387357 PMC4417576 25960750

[20] Y. C. Wu , G. X. Wu , H. H. Huang , and S. M. Kuo , “ Liposome-encapsulated farnesol accelerated tissue repair in third-degree burns on a rat model,” Burns 45(5), 1139–1151 (2019). 10.1016/j.burns.2019.01.010 30833099

[21] Y. H. Lin , S. I. Lee , F. H. Lin , G. X. Wu , C. S. Wu , and S. M. Kuo , “ Enhancement of rotator cuff healing with farnesol-impregnated gellan gum/hyaluronic acid hydrogel membranes in a rabbit model,” Pharmaceutics 13(7), 944 (2021). 10.3390/pharmaceutics13070944 34202556 PMC8309098

[22] G. X. Wu , C. Y. Chen , C. S. Wu , L. C. Hwang , S. W. Yang , and S. M. Kuo , “ Restoration of the phenotype of dedifferentiated rabbit chondrocytes by sesquiterpene farnesol,” Pharmaceutics 14(1), 186 (2022). 10.3390/pharmaceutics14010186 35057081 PMC8779926

[23] C. Colombo , M. Butler , E. O'Byrne *et al.*, “ A new model of osteoarthritis in rabbits. I. Development of knee joint pathology following lateral meniscectomy and section of the fibular collateral and sesamoid ligaments,” Arthritis Rheum. 26(7), 875–886 (1983). 10.1002/art.1780260709 6688183

[24] Y. Yoshihara , H. Nakamura , K. Obata *et al.*, “ Matrix metalloproteinases and tissue inhibitors of metalloproteinases in synovial fluids from patients with rheumatoid arthritis or osteoarthritis,” Ann. Rheum. Dis. 59(6), 455–461 (2000). 10.1136/ard.59.6.455 10834863 PMC1753174

[25] H. Wu , J. Du , and Q. Zheng , “ Expression of MMP-1 in cartilage and synovium of experimentally induced rabbit ACLT traumatic osteoarthritis: Immunohistochemical study,” Rheumatol. Int. 29(1), 31–36 (2008). 10.1007/s00296-008-0636-2 18597092

[26] J. W. Alford and B. J. Cole , “ Cartilage restoration, part 1: Basic science, historical perspective, patient evaluation, and treatment options,” Am. J. Sports Med. 33(2), 295–306 (2005). 10.1177/0363546504273510 15701618

[27] H. P. Gerber , T. H. Vu , A. M. Ryan , J. Kowalski , Z. Werb , and N. Ferrara , “ VEGF couples hypertrophic cartilage remodeling, ossification and angiogenesis during endochondral bone formation,” Nat. Med. 5(6), 623–628 (1999). 10.1038/9467 10371499

[28] D. A. Krawczak , J. J. Westendorf , C. S. Carlson , and J. L. Lewis , “ Influence of bone morphogenetic protein-2 on the extracellular matrix, material properties, and gene expression of long-term articular chondrocyte cultures: Loss of chondrocyte stability,” Tissue Eng., Part A 15(6), 1247–1255 (2009). 10.1089/ten.tea.2008.0249 18950256 PMC2792092

[29] S. Suri and D. A. Walsh , “ Osteochondral alterations in osteoarthritis,” Bone 51(2), 204–211 (2012). 10.1016/j.bone.2011.10.010 22023932

[30] A. Haseeb , R. Kc , M. Angelozzi *et al.*, “ SOX9 keeps growth plates and articular cartilage healthy by inhibiting chondrocyte dedifferentiation/osteoblastic redifferentiation,” Proc. Natl. Acad. Sci. U. S. A. 118(8), e2019152118 (2021). 10.1073/pnas.2019152118 33597301 PMC7923381

[31] Q. Zhang , Q. Ji , X. Wang *et al.*, “ SOX9 is a regulator of ADAMTSs-induced cartilage degeneration at the early stage of human osteoarthritis,” Osteoarthritis Cartilage 23(12), 2259–2268 (2015). 10.1016/j.joca.2015.06.014 26162802

[32] N. G. M. Thielen , P. M. van der Kraan , and A. P. M. van Caam , “ TGFβ/BMP signaling pathway in cartilage homeostasis,” Cells 8(9), 969 (2019). 10.3390/cells8090969 31450621 PMC6769927

[33] G. Coricor and R. Serra , “ TGF-β regulates phosphorylation and stabilization of SOX9 protein in chondrocytes through p38 and Smad dependent mechanisms,” Sci Rep 6, 38616 (2016). 10.1038/srep38616 27929080 PMC5144132

[34] R. D. Chavez , G. Coricor , J. Perez , H. S. Seo , and R. Serra , “ SOX9 protein is stabilized by TGF-β and regulates PAPSS2 mRNA expression in chondrocytes,” Osteoarthritis Cartilage 25(2), 332–340 (2017). 10.1016/j.joca.2016.10.007 27746378 PMC5258840

[35] L. J. Sandell and T. Aigner , “ Articular cartilage and changes in arthritis. An introduction: Cell biology of osteoarthritis,” Arthritis Res. 3(2), 107–113 (2001). 10.1186/ar148 11178118 PMC128887

[36] F. Wei , J. Zhou , X. Wei *et al.*, “ Activation of Indian hedgehog promotes chondrocyte hypertrophy and upregulation of MMP-13 in human osteoarthritic cartilage,” Osteoarthritis Cartilage 20(7), 755–763 (2012). 10.1016/j.joca.2012.03.010 22469853 PMC3374008

[37] Z. Li , B. Liu , D. Zhao , B. Wang , Y. Liu , Y. Zhang , F. Tian , and B. Li , “ Protective effects of nebivolol against interleukin-1beta (IL-1beta)-induced type II collagen destruction mediated by matrix metalloproteinase-13 (MMP-13),” Cell Stress Chaperones 22, 767–774 (2017). 10.1007/s12192-017-0805-x 28512729 PMC5655365

[38] H. Cho , A. Walker , J. Williams , and K. A. Hasty , “ Study of osteoarthritis treatment with anti-inflammatory drugs: Cyclooxygenase-2 inhibitor and steroids,” Biomed. Res. Int. 2015, 595273. 10.1155/2015/595273 26000299 PMC4427003

[39] R. C. Gupta , R. Lall , A. Srivastava , and A. Sinha , “ Hyaluronic acid: Molecular mechanisms and therapeutic trajectory,” Front. Vet. Sci. 6, 192 (2019). 10.3389/fvets.2019.00192 31294035 PMC6603175

[40] D. Needham , A. Arslanagic , K. Glud *et al.*, “ Bottom up design of nanoparticles for anti-cancer diapeutics: ‘Put the drug in the cancer's food,’” J. Drug Target 24(9), 836–856 (2016). 10.1080/1061186X.2016.1238092 27646195

[41] Y. J. Wu , Y. C. Wu , I. F. Chen *et al.*, “ Reparative effects of astaxanthin-hyaluronan nanoaggregates against retrorsine-CCl_4_-induced liver fibrosis and necrosis,” Molecules 23(4), 726 (2018). 10.3390/molecules23040726 29565318 PMC6017246

[42] A. Matsiko , T. J. Levingstone , F. J. O'Brien , and J. P. Gleeson , “ Addition of hyaluronic acid improves cellular infiltration and promotes early-stage chondrogenesis in a collagen-based scaffold for cartilage tissue engineering,” J. Mech. Behav. Biomed. Mater. 11, 41–52 (2012). 10.1016/j.jmbbm.2011.11.012 22658153

[43] L. C. Lin , S. J. Chang , C. Y. Lin *et al.*, “ Repair of chondral defects with allogenous chondrocyte-seeded hyaluronan/collagen II microspheres in a rabbit model,” Artif. Organs 36(4), E102–E109 (2012). 10.1111/j.1525-1594.2011.01370.x 22145763

[44] A. K. Grover and S. E. Samson , “ Benefits of antioxidant supplements for knee osteoarthritis: Rationale and reality,” Nutr. J. 15, 1 (2016). 10.1186/s12937-015-0115-z 26728196 PMC4700773

